# PFAS and One Health: integrating human, animal, and environmental perspectives

**DOI:** 10.1007/s00204-026-04306-1

**Published:** 2026-02-17

**Authors:** Marília Cristina Oliveira Souza, Fernando Barbosa, Jose L. Domingo

**Affiliations:** 1 School of Pharmaceutical Sciences of Ribeirão Preto, Department of Biomolecular Sciences, University of São Paulo, Av. do Café s/n, Ribeirao Preto, SP 14040-903 Brazil; 2 School of Pharmaceutical Sciences of Ribeirão Preto, Department of Clinical, Toxicological and Bromatological Analysis, University of São Paulo, Ribeirão Preto, SP Brazil; 3 Laboratory of Toxicology and Environmental Health, School of Medicine, Universitat Rovira I Virgili, Sant Llorenç 21, 43201 Reus, Catalonia Spain

**Keywords:** PFAS, One Health, Environmental health, Bioaccumulation, Regulation

## Abstract

Per- and polyfluoroalkyl substances (PFAS)—ubiquitous, persistent, and bioaccumulative—pose a cross-cutting threat to human, animal, and environmental health. Their resistance to degradation and global dissemination demand an integrated One Health framework to address their complex, interdependent risks across ecosystems, wildlife, and public health. This review synthesizes current knowledge on PFAS, highlighting their anthropogenic origins, environmental persistence, and global dispersion. Common exposure pathways, such as dietary intake, contaminated drinking water, and maternal transfer, lead to convergent toxicological outcomes across species, including immunotoxicity, reproductive and developmental impairments, and carcinogenicity, validating the use of cross-species data in risk assessment and reinforcing the need for integrated protective strategies. Vulnerable human populations and wildlife face disproportionate risks, with the latter acting as both sentinels and exposure sources. Despite regulatory efforts, the ongoing use and development of replacement compounds sustain PFAS as a global concern. Effectively addressing this challenge requires a coordinated, cross-sectoral strategy that integrates environmental monitoring, transdisciplinary research, and harmonized regulatory frameworks. The One Health paradigm provides the essential model for developing these collaborative solutions.

## Introduction

### Per-and polyfluoroalkyl substances (PFAS)

Per-and polyfluoroalkyl substances (PFAS) are synthetic organofluorine compounds that have been extensively utilized in industrial and consumer applications for decades due to their exceptional thermal and chemical stability, surfactant properties, and resistance to degradation (Lindstrom et al. 2011; Prevedouros et al. 2006; Rahman et al. 2014; Wang et al. 2017). These compounds represent a paradigmatic environmental health challenge that transcends traditional disciplinary boundaries, compelling innovative approaches that embrace the fundamental interconnectedness of human, animal, and environmental health systems (Buck et al. 2011; Glüge et al. 2020; OECD 2021). However, these same unique properties have resulted in their emergence as one of the most pervasive and persistent environmental contaminants (Cousins et al. 2020; Evich et al. 2022). The PFAS family includes over 14,000 substances with diverse chemical structures and properties. Long-chain compounds typically show higher bioaccumulation and longer human half-lives, whereas short-chain variants are characterized by increased environmental mobility despite similar persistence. Toxicological impacts also differ; while PFOS and PFOA are associated with hepatotoxic and developmental risks, newer or shorter-chain alternatives may operate through different biological mechanisms (Bil et al. 2021; Fenton et al. 2021).

PFAS are ubiquitously detected in environmental compartments worldwide, including remote Arctic and Antarctic regions, demonstrating their capacity for long-range transport and global distribution (Butt et al. 2010; Casal et al. 2017). The carbon–fluorine bond, one of the strongest chemical bonds known, renders these compounds virtually indestructible under normal environmental conditions, with atmospheric half-lives ranging from decades to millennia (Cousins et al. 2020). Nearly 5,000 PFAS have been identified in commercial use and environmental samples, with this number continuing to grow (OECD 2021; Wang et al. 2017; Zhara et al. 2025). Legacy PFAS, such as perfluorooctanoic acid (PFOA) and perfluorooctane sulfonic acid (PFOS), have been the focus of extensive research and regulatory action under international agreements such as the Stockholm Convention on Persistent Organic Pollutants (Stockholm Convention, 2019). Simultaneously, the introduction of newer PFAS compounds as replacements for legacy chemicals creates a complex and evolving contamination landscape (Cousins et al. 2020; Kwiatkowski et al. 2020).

### The compelling case for one health application

The One Health concept recognizes that the health of humans, domestic and wild animals, and the wider environment are inextricably linked and interdependent, providing a crucial framework for understanding and addressing PFAS challenges (CDC, 2024; Zinsstag et al. 2011). This approach has evolved from its origins in veterinary medicine to encompass broader environmental health challenges, where contamination affects multiple species and ecosystems simultaneously (Domingo et al. 2025; Nguyen-Viet et al. 2025). PFAS contamination presents an ideal case study for One Health principles due to several intrinsic characteristics that highlight fundamental interconnections between environmental, animal, and human health domains (Destoumieux-Garzón et al. 2018; Lerner and Berg 2017).

PFAS exposure results in remarkably similar adverse health outcomes across diverse species, including immunosuppression, reproductive and developmental toxicity, liver dysfunction, and potential carcinogenic effects (DeWitt et al. 2019; Fair et al. 2019; Pesonen and Vähäkangas 2024). This convergence reflects shared toxicological mechanisms and validates the use of cross-species extrapolation in risk assessment. The immunotoxic effects of PFAS have been documented across numerous species from laboratory rodents to marine mammals to human populations, with consistent patterns of reduced antibody responses, altered immune cell function, and increased susceptibility to infectious diseases (Bline et al. 2024; Houde et al. 2011; Steenland et al. 2018).

Food webs serve as the primary conduit for PFAS transfer and bioaccumulation, with wildlife populations acting as both sentinels for environmental contamination and sources of human exposure through consumption of contaminated animal products (Houde et al. 2011; Michaud et al. 2025). This creates direct linkages between ecosystem health and human health that transcend traditional environmental and public health boundaries. Bioaccumulation patterns vary among PFAS compounds, with longer-chain compounds showing greater bioaccumulation potential, while shorter-chain replacement compounds may be more mobile and widespread in environmental media (Gkika et al. 2025).

### Objectives and structure

**Fig. 1:**
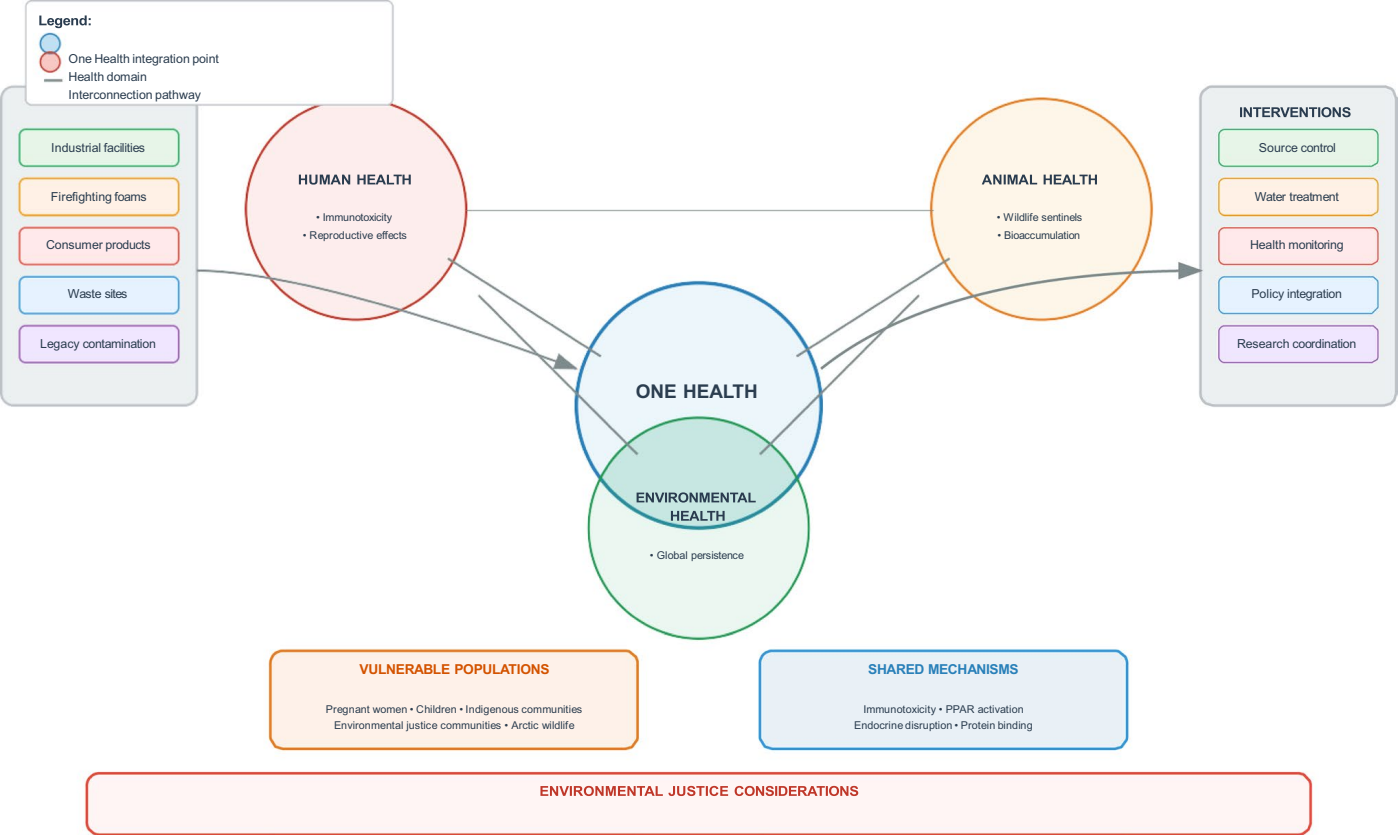
A one health conceptual framework for PFAS contamination

This review is aimed at synthesizing the current knowledge on PFAS contamination through an explicit One Health lens, examining interconnected sources, fate, exposure pathways, and health effects across human, animal, and environmental domains. The specific objectives are the following: (1) to characterize shared sources and environmental fate processes creating global contamination patterns, (2) to evaluate convergent health impacts across human and wildlife populations, (3) to identify critical interconnections and shared vulnerabilities linking the three health domains, (4) to analyze environmental justice considerations in PFAS exposure, (5) to propose integrated intervention strategies based on One Health principles, and to (6) highlight knowledge gaps requiring transdisciplinary collaboration. The conceptual framework for applying One Health principles to PFAS management, illustrating the interconnected sources, health domains, intervention strategies, and vulnerable populations that must be addressed through coordinated action, is depicted in Fig. 1.

### Methods (search strategy)

A comprehensive literature review was conducted to synthesize existing evidence on PFAS within the One Health framework, including publications until October 31, 2025. This timeframe captures the emergence of PFAS as a global environmental health concern and the evolution of One Health as a framework for addressing complex environmental challenges. The search strategy was designed to identify those studies demonstrating interconnections between human health, animal health, and environmental health aspects related to PFAS contamination, exposure, and effects.

Databases used were PubMed (https://pubmed.ncbi.nlm.nih.gov), Web of Science (https://www.webofscience.com), Scopus (https://www.scopus.com), and Google Scholar (https://scholar.google.com.br). A set of search terms and their combinations was employed using Boolean operators (AND, OR): "per- and polyfluoroalkyl substances" OR “PFAS” OR “perfluoroalkyl” OR “polyfluoroalkyl”; “PFOA” OR “perfluorooctanoic acid” OR “PFOS” OR "perfluorooctane sulfonic acid" OR “GenX” OR “PFNA” OR “PFHxS”; “One Health” OR “One Medicine” OR “EcoHealth” OR “Planetary Health”; “environmental health” OR “ecotoxicology” OR “wildlife toxicology”; “bioaccumulation” OR “biomagnification” OR "persistent organic pollutant"; “exposure pathway” OR “human exposure” OR “wildlife exposure”; “immunotoxicity” OR “reproductive toxicity” OR “developmental toxicity” OR “carcinogenicity”; “risk assessment” OR “regulation” OR “management” OR “remediation”; “environmental justice” OR "vulnerable populations."

Studies were included if they were focused primarily on PFAS or specific PFAS compounds, demonstrated clear relevance to at least two domains of the One Health triad, reported original data, systematic reviews, meta-analyses, or significant conceptual advances linking these domains. Scientific literature from international organizations (UNEP, WHO, OECD, US EPA, ECHA) was included to supplement peer-reviewed sources. The selected literature was analyzed through a structured One Health framework considering interconnections between environmental contamination, wildlife health, and human health, identifying: (a) shared sources and exposure pathways, (b) convergent toxicological mechanisms and health effects across species, (c) feedback loops and interconnections between health domains, (d) vulnerable populations and environmental justice considerations, (e) integrated intervention strategies with co-benefits across domains, and (f) critical knowledge gaps requiring transdisciplinary research approaches.

## Results

### PFAS sources, environmental fate, and shared exposure pathways

PFAS contamination originates from various human-made sources, establishing common pathways of exposure that are linked to multiple health outcomes (De Silva et al. 2021; Fenton et al. 2021; Sunderland et al. 2019). Industrial manufacturing represents the most significant historical source, with major production facilities creating severe contamination hotspots affecting local communities, wildlife habitats, and water resources simultaneously (Hu et al. 2016; Sadia et al. 2023; Schultz et al. 2004). The legacy of industrial production has been particularly evident in areas such as the Ohio River Valley, where decades of PFOA production contaminated drinking water supplies for hundreds of thousands of people, while affecting aquatic ecosystems throughout the region (Herrick et al. 2017). Firefighting activities represent another major source category, with aqueous film-forming foams (AFFF) containing PFAS having been used extensively at military bases, airports, and industrial facilities since the 1960s (Mazumder et al. 2023; Moody et al. 2002, 2003). In relation to this, the US Department of Defense has identified approximately 700 military installations with known or suspected PFAS contamination, affecting millions of people and vast areas of natural habitat (US GAO 2021).

Consumer products represent a more diffuse but globally significant source category, with PFAS-containing products including non-stick cookware, water-resistant textiles, food packaging, and personal care products contributing to global contamination through use, disposal, and breakdown (Domingo 2012; Glüge et al. 2020; Yang et al. 2025). The ubiquity of consumer products means that virtually every household in developed countries contains multiple PFAS sources, creating widespread indoor contamination affecting both human occupants and domestic animals (Sunderland et al. 2019). On the other hand, incineration of PFAS-containing materials can create new fluorinated compounds through thermal decomposition processes, while waste-to-energy facilities may volatilize and redistribute PFAS through air emissions (Weitz et al. 2024).

### Environmental fate and transport

**Fig. 2:**
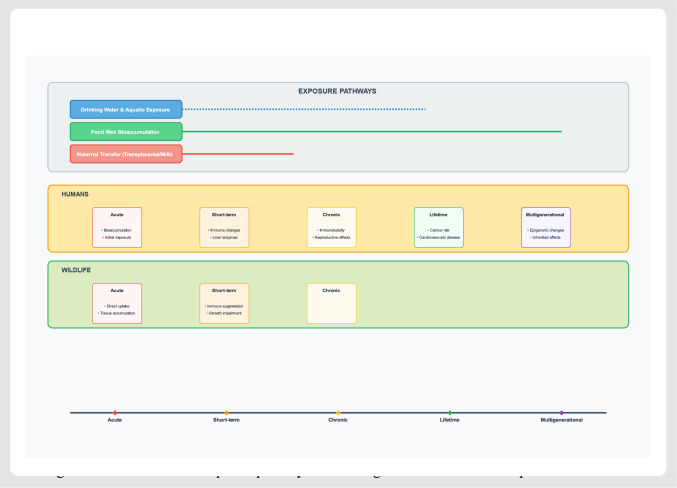
Timeline of PFAS exposure pathways and convergent health effects across species

The extreme persistence of PFAS stems from the strength of the carbon–fluorine bond, rendering these compounds resistant to virtually all environmental degradation processes (Cousins et al. 2020; Walker and Milligan 2025). This persistence ensures that contamination, once established, will affect multiple generations of humans and wildlife, creating essentially permanent contamination that requires active management rather than natural attenuation. Global distribution occurs through atmospheric transport, oceanic transport through surface currents, and freshwater systems serving as major conduits for PFAS transport (Butt et al. 2010; Casal et al. 2017). Environmental partitioning behaviors vary significantly among PFAS compounds, resulting in distinct yet interconnected exposure scenarios. Figure 2 depicts the temporal progression of PFAS exposure pathways and health effects across species, demonstrating how different exposure routes (drinking water, food web bioaccumulation, and maternal transfer) lead to convergent health outcomes in both humans and wildlife over time scales ranging from acute exposure to multigenerational effects.

Long-chain PFAS (C8 and longer) tend to partition to sediments and biota due to their greater hydrophobicity, leading to bioaccumulation and biomagnification through food webs. In turn, short-chain PFAS (C4-C7) remain highly mobile in water systems, creating direct human exposure pathways through drinking water contamination (Zhao et al. 2016). Bioaccumulation and biomagnification patterns create direct linkages between environmental contamination and exposure in both wildlife and humans consuming contaminated animals (Houde et al. 2011; Kelly et al. 2009). PFAS bioaccumulation differs from the traditional Persistent Organic Pollutants (POPs) in that it occurs primarily through protein binding rather than lipid partitioning, leading to accumulation in blood, liver, and kidney tissues (Domingo 2025; Kärrman et al. 2010; Pérez et al. 2013).

Climate change introduces additional complexity to PFAS environmental fate through multiple mechanisms affecting all health domains (Liu and Mejia Avendaño, 2013; Weber et al. 2017). Changing precipitation patterns may alter the hydrological cycling of PFAS, while rising temperatures may enhance the volatilization of certain compounds. Arctic environments face vulnerabilities such as melting sea ice that may alter PFAS partitioning and bioavailability in marine ecosystems (Arulananthan et al. 2025; Lukic Bilela et al. 2023).

The interconnected sources, exposure pathways, and health outcomes of PFAS across human, animal, and environmental domains are presented in Table 1. This integrated perspective shows how shared contamination pathways propagate risks through all tiers of the One Health triad, reinforcing the necessity for cross-sectoral interventions.

**Table 1:** Major sources, exposure pathways, and health effects of PFAS across one health domains

Domain	Main sources/reservoirs	Principal exposure pathways	Key adverse health effects	Vulnerable groups/species
Human	Industrial facilities, firefighting foam sites, consumer products, contaminated water supplies, food packaging	Drinking water, dietary intake (fish, meat, food packaging), occupational exposure, maternal transfer, consumer products	Immunotoxicity, reproductive impairment, developmental toxicity, liver dysfunction, carcinogenicity, cardiovascular effects	Pregnant women, children, communities near industrial sites, firefighters, chemical workers, indigenous communities
Wildlife	Contaminated water bodies, sediments, prey species, maternal transfer, bioaccumulation hotspots	Ingestion (prey, water), sediment contact, maternal transfer (eggs, lactation), bioaccumulation through food webs	Immunosuppression, reproductive failure, developmental abnormalities, endocrine disruption, liver toxicity, population-level effects	Apex predators (marine mammals, birds of prey), aquatic organisms, Arctic wildlife, long-lived species, developing offspring
Environment	Industrial releases, landfills, wastewater treatment plants, atmospheric transport, legacy contamination	Environmental partitioning (water, soil, sediments, air), long-range transport, extreme persistence, global distribution	Ecosystem contamination, food web accumulation, global distribution, permanent environmental burden, aquifer contamination	Aquatic ecosystems, Arctic environments, contaminated sites, groundwater systems, watersheds near industrial sources

### Human health impacts: pathways and effects

Human exposure to PFAS occurs through multiple interconnected pathways, reflecting the ubiquity of these compounds and creating exposure scenarios that parallel those experienced by wildlife populations (Domingo and Nadal 2019; Sunderland et al. 2019).

#### Exposure pathways

Drinking water represents a major exposure source for millions of people worldwide, particularly in communities near industrial sources or military installations (Hu et al. 2016; Post et al. 2017; Post 2021; Steenland et al. 2018). Recent surveys indicate detectable levels of PFAS in drinking water supplies serving over 160 million Americans (US Environmental Working Group, 2025). Private wells may pose risks to rural populations that lack adequate monitoring and treatment infrastructure. In turn, dietary intake contributes significantly through multiple food categories reflecting environmental distribution and bioaccumulation (Domingo 2012; Domingo and Nadal 2019; Melnyk et al. 2025; Souza and Domingo 2025). Seafood consumption represents a major source due to bioaccumulation in aquatic food webs (Domingo 2012; Domingo et al. 2012), while meat and dairy products can contain PFAS due to contamination of animal feed or grazing areas (Bonato et al. 2025; Ericson et al. 2008; Hossini et al. 2025). Food packaging applications create direct exposure pathways through migration of PFAS from packaging materials to food (Ericson et al. 2008).

Occupational exposure represents the highest exposure scenario for certain worker populations, including those in PFAS manufacturing, firefighting, and semiconductor production (Alexander and Olsen 2007; Grice et al. 2007). Firefighters face particularly high exposures due to PFAS-containing firefighting foams and the presence of PFAS in smoke from modern materials (Mazumder et al. 2023; Rotander et al. 2024).

Maternal transfer creates critical exposure pathways during vulnerable developmental periods, with PFAS readily crossing the placental barrier and being transferred through breast milk (Fenton et al. 2009; González and Domingo 2025; Mamsen et al. 2019). The long half-lives of PFAS mean that maternal body burdens accumulated over years are transferred to infants, creating intergenerational effects.

#### Human health effects

The health effects of PFAS exposure demonstrate remarkable consistency across different populations, suggesting common mechanisms of toxicity (DeWitt et al. 2019; Fenton et al. 2021; Mahoney et al. 2022). Immunotoxicity represents one of the most robust effects, with consistent evidence demonstrating that PFAS can suppress immune function and increase susceptibility to infectious diseases (Bline et al. 2024; DeWitt et al. 2019; Grandjean et al. 2012). Studies have shown reduced antibody responses to vaccines, increased frequency of infections, and altered immune cell populations at environmentally relevant exposure levels (Qiu, 2025).

Reproductive and developmental toxicity represents another well-established effect of PFAS, with evidence from both human and animal studies demonstrating impacts on fertility, pregnancy outcomes, and child development (Bach et al. 2015; Steenland et al. 2018). PFAS concentrations have been associated with adverse effects, including reduced fertility, altered hormone levels, reduced birth weight, preterm birth, intrauterine growth restriction, preeclampsia, delayed puberty, and behavioral effects that may persist into adulthood. In turn, liver toxicity and metabolic disruption are consistently observed across species and exposure scenarios (Gallo et al. 2012; Steenland et al. 2018; Souza et al. 2020). Moreover, human studies have documented elevated liver enzymes, altered lipid profiles, and increased risk of diabetes and metabolic syndrome (Kashobwe et al. 2024; Zhang et al. 2023).

On the other hand, carcinogenic potential has been reported for certain PFAS compounds, with PFOA classified as carcinogenic to humans, based on sufficient evidence for kidney and testicular cancer (Steenland and Winquist 2021; Stevenson et al. 2021). Cardiovascular effects have also emerged as an area of increasing concern, with epidemiological studies suggesting associations with hypertension and cardiovascular disease (Odediran et al. 2025).

#### Vulnerable populations and health disparities

PFAS exposure demonstrates significant disparities across populations, with certain groups facing disproportionately high exposures or enhanced susceptibility (Hu et al. 2016). Pregnant women and children represent populations of particular concern due to developmental toxicity and exposure during critical windows of development (Fenton et al. 2009; Mamsen et al. 2019; Souza et al. 2020). Children may face higher exposures due to greater food and water intake per unit body weight and behaviors that increase exposure.

Communities near industrial sources face some of the highest documented PFAS exposures, with blood levels that can be orders of magnitude higher than background populations (Hu et al. 2016; Steenland et al. 2018). These communities often include environmental justice populations who face additional vulnerabilities due to socioeconomic factors and limited access to healthcare. Indigenous and subsistence communities face unique vulnerabilities due to their reliance on traditional foods that may bioaccumulate PFAS, particularly in Arctic environments (Butt et al. 2010). These communities face the difficult choice between maintaining traditional food systems central to their cultural identity and avoiding exposure.

### Wildlife health impacts: sentinels and shared vulnerabilities

Wildlife populations serve as critical environmental sentinels, providing early warnings of emerging threats, while representing direct conservation concerns (Fair et al. 2019; Houde et al. 2011).

#### Exposure pathways and bioaccumulation

Due to their direct exposure to contaminated water and position at the base of aquatic food webs, aquatic organisms represent the most extensively studied wildlife group (Kelly et al. 2009; Lukić Bilela et al. 2023; Martin et al. 2004). Fish accumulate PFAS through multiple pathways, including direct uptake from water, ingestion of contaminated food, and sediment contact. Unlike traditional lipophilic contaminants, PFAS accumulate primarily in protein-rich tissues such as blood, liver, and muscle. Marine mammals represent apex predators that often exhibit the highest PFAS concentrations recorded in wildlife, with levels that can exceed those in heavily exposed human populations (Butt et al. 2010; Fair et al. 2019). Arctic marine mammals face special risks due to their reliance on marine food webs receiving PFAS inputs from global transport processes (Lee et al. 2023).

Maternal transfer represents a critical exposure pathway across all taxonomic groups, with PFAS readily transferred through eggs in oviparous species and through placental transfer and lactation in mammals (Fair et al. 2019; Ricolfi et al. 2024). This creates multigenerational effects linking adult exposure to offspring health outcomes.

#### Wildlife health effects

Marine mammals demonstrate some of the clearest evidence for PFAS-induced health effects in wildlife, with documented impacts on immune function, reproductive success, and survival (Butt et al. 2010; Fair et al. 2019). Immunotoxic effects include reduced antibody responses and increased susceptibility to infectious diseases, paralleling effects observed in human populations (Lukić Bilela et al. 2023). In turn, seabirds serve as excellent sentinels for marine PFAS contamination due to their high trophic position and wide geographic distribution (Bustnes et al. 2007; Nordstad et al. 2012). Health effects include altered immune function, reduced reproductive success, and behavioral changes affecting survival. Fish and aquatic invertebrates show a range of PFAS-induced effects, including developmental abnormalities, behavioral changes, and reproductive impairment (Keiter et al. 2012; MacDonald et al. 2004). Laboratory studies have demonstrated that environmentally relevant PFAS concentrations can cause developmental defects and disrupt endocrine function (Banyoi et al. 2022).

#### Ecosystem-level effects

The impacts of PFAS on wildlife extend beyond individual organisms to affect population dynamics, community structure, and ecosystem function (Fair et al. 2019; Houde et al. 2011). Population-level effects may occur when individual impacts on survival or reproduction translate into changes in population growth rates. The role of wildlife as PFAS sentinels provides critical information for protecting both ecosystem and human health, with wildlife monitoring detecting contamination problems before they are observed in humans.

### Shared mechanisms of toxicity: convergent biological vulnerabilities

The consistency of adverse health effects across diverse species reflects shared toxicological mechanisms and conserved biological vulnerabilities (DeWitt et al. 2019; Lau et al. 2007).

#### Immune system disruption

PFAS-induced immunotoxicity represents one of the most consistent effects across species, with similar mechanisms and outcomes observed in laboratory animals, wildlife, and human populations (DeWitt et al. 2019; Fairley et al. 2007; Qiu et al. 2025). PFAS interfere with immune function through multiple mechanisms, including altered cytokine production, impaired antibody responses, and disruption of immune cell development. Molecular mechanisms involve disruption of signaling pathways conserved across vertebrate species (Qiu et al. 2025).

#### Endocrine disruption and developmental toxicity

PFAS act as endocrine disruptors through multiple mechanisms, affecting hormone synthesis, transport, and signaling pathways that are highly conserved across vertebrate species (Ballesteros et al. 2017; Bulawska et al. 2025; Chang et al. 2008). Thyroid hormone disruption is particularly well-documented, with PFAS interfering with thyroid hormone transport proteins and metabolism (Sosnowska et al. 2025). The developmental toxicity of PFAS stems from their ability to disrupt critical developmental processes during embryonic and fetal development, with effects that can be permanent and irreversible (Fenton et al. 2021).

#### Peroxisome proliferator-activated receptor (PPAR) activation

Many PFAS compounds activate PPARα, a nuclear receptor regulating gene expression involved in lipid metabolism and inflammatory responses (Lau et al. 2007; Wolf et al. 2008). PPAR activation can lead to hepatocellular effects, including hepatomegaly and altered lipid metabolism. While there are species differences in PPAR responsiveness, these pathways are conserved across vertebrate species (Beccacece et al. 2023; Cheng et al. 2021).

#### Oxidative stress and cellular damage

PFAS exposure can induce oxidative stress and inflammatory responses across diverse cell types and species, leading to cellular damage that may contribute to observed toxic effects (Eriksen et al. 2010; Shi et al. 2007). Chronic inflammation is associated with numerous diseases and may provide a mechanistic link between PFAS exposure and health outcomes (Chen et al. 2024).

### Environmental justice and vulnerable populations

The distribution of PFAS contamination reveals striking patterns of environmental injustice, highlighting intersections between environmental degradation, social inequality, and health disparities (Hu et al. 2016). PFAS contamination exhibits clear geographic patterns that reflect both the locations of contamination sources and the distribution of vulnerable populations (Hu et al. 2016; Steenland et al. 2018). Industrial contamination sources are often located near communities of color and low-income communities, reflecting historical environmental racism. Military installations, representing major PFAS contamination sources (Ruyle et al. 2023), are often located near communities that depend on the military economically but bear environmental health costs. Rural communities face specific vulnerabilities due to reliance on private wells for drinking water and limited access to treatment infrastructure (Hu et al. 2016; Kosiarski et al. 2025). Agricultural communities may face additional exposures through contaminated irrigation water and biosolids application. Urban environmental justice communities often face cumulative exposures to PFAS and other contaminants, creating compound health risks (Brunn et al. 2023). Environmental justice communities often face multiple environmental stressors that can interact with PFAS exposure to create cumulative health risks exceeding those from any single source (Brunn et al. 2023). These stressors may include other chemical contaminants, air pollution, and social stressors such as poverty and inadequate healthcare access.

### Integrated intervention strategies: one health solutions

Effectively addressing PFAS contamination requires integrated strategies considering impacts across all health domains while seeking solutions that generate co-benefits (Cousins et al. 2020; Kwiatkowski et al. 2020). Table 2 shows integrated One Health strategies for PFAS management, detailing source control, technological remediation, policy innovations, and their cross-domain co-benefits. This framework highlights the synergies achievable when interventions simultaneously address human, animal, and environmental health dimensions.

**Table 2:** Integrated one health intervention strategies for PFAS management

Strategy type	Description	Expected co-benefits across domains	Implementation challenges
Source control and regulation	International agreements (Stockholm Convention), national restrictions, essential use frameworks, class-based regulation, safer alternatives development	Reduces new emissions globally, protects human health, wildlife, and ecosystems from ongoing contamination, prevents regrettable substitutions	Defining essential uses, developing alternatives, international coordination, industry resistance
Water treatment and remediation	Advanced treatment technologies (activated carbon, ion exchange, membrane filtration), soil stabilization, sediment management, destruction technologies	Improves drinking water quality, reduces aquatic ecosystem exposure, protects groundwater resources, reduces bioaccumulation	High costs, energy requirements, waste management, technology limitations for short-chain PFAS
Exposure reduction	Drinking water standards, dietary advisories, product restrictions, occupational protections, consumer education	Directly reduces human exposure while protecting wildlife through reduced environmental releases, market transformation	Enforcement challenges, consumer acceptance, economic impacts, technical performance requirements
Integrated surveillance	Coordinated monitoring of PFAS in water, wildlife, food, and human biomonitoring programs, total organofluorine measurements	Enables early detection, tracks intervention effectiveness, identifies emerging hotspots across all domains, supports adaptive management	Analytical costs, method limitations, data integration, sustained funding requirements
Waste management	Improved landfill design, wastewater treatment upgrades, proper disposal of PFAS-containing products, waste minimization	Reduces legacy sources, protects both communities and ecosystems near waste facilities, prevents cross-media transfer	Infrastructure costs, technology limitations, waste characterization, liability issues
Environmental justice	Targeted interventions for high-exposure communities, cumulative risk assessment, community engagement, health equity measures	Promoting environmental justice and biodiversity conservation simultaneously, addresses underlying vulnerabilities	Resource requirements, community capacity, institutional barriers, complex cumulative effects
Research and innovation	Transdisciplinary research, alternatives development, remediation technology advancement, analytical method development	Fosters innovation benefiting all domains, improves risk assessment and management strategies, supports evidence-based policy	Funding sustainability, technology transfer, interdisciplinary coordination, commercial viability
Policy integration	Harmonized regulations, cross-sectoral collaboration, international cooperation, adaptive management frameworks	Ensures comprehensive, effective PFAS management with global coordination, prevents regulatory gaps	Institutional barriers, sovereignty issues, economic competition, varying regulatory capacities

### Source control and regulation

Global source control represents the most fundamental approach to PFAS management, requiring coordinated international action to phase out non-essential uses while maintaining critical applications where no viable alternatives exist (Cousins et al. 2019; Stockholm Convention, 2019). The Stockholm Convention listing of PFOA, PFOS, and related compounds provides a framework for international cooperation. The essential use concept provides a framework for distinguishing between PFAS applications critical for societal functions and those that could be eliminated.

Regulatory restrictions at national and regional levels have demonstrated potential for reducing PFAS emissions and environmental concentrations (ECHA, 2023; US EPA, 2024). The European Union’s proposed restriction on PFAS as a class represents the most comprehensive regulatory approach to date. The development of safer alternatives to PFAS requires significant investment in research and development, as well as regulatory frameworks encouraging innovation while ensuring alternatives do not create new problems (Glüge et al. 2020).

#### Water treatment and environmental remediation

Advanced water treatment technologies provide effective approaches for removing PFAS from drinking water and wastewater, although costs and technical requirements present implementation challenges (Dixit et al. 2021; Jafarinejad 2025; Ross et al. 2018). Activated carbon treatment is effective for longer-chain PFAS but may be less effective for shorter-chain compounds. Ion exchange resins and membrane technologies can achieve high removal efficiencies, although they require significant energy inputs and produce concentrated waste streams requiring proper management (Jafarinejad 2025; Liu and Sun 2021).

Soil and sediment remediation for PFAS is challenging due to strong binding to soil particles and lack of effective in-situ treatment technologies (Weber et al. 2017). Excavation and ex-situ treatment can be effective but is expensive and disruptive. Watershed-scale management approaches considering PFAS sources, transport, and fate across entire watersheds provide opportunities for comprehensive contamination control benefiting both human and ecological health (Sunderland et al. 2019).

#### Public health interventions

Public health interventions including biomonitoring, health surveillance, and medical monitoring can provide early detection of PFAS-related health effects and guide treatment decisions (Rogers et al. 2021; Steenland et al. 2018). Biomonitoring programs can track exposure trends and identify populations with elevated exposures. Dietary exposure reduction strategies include fish consumption advisories, food safety standards, and agricultural management practices reducing PFAS uptake in crops and livestock (Bonato et al. 2025; Shurson 2025).

#### Integrated surveillance and monitoring

Comprehensive environmental monitoring programs tracking PFAS across environmental compartments provide essential information for understanding contamination sources and trends (Dimitrakopoulou et al. 2024; Houde et al. 2011). Wildlife biomonitoring programs provide early warning of environmental contamination and can detect emerging problems before they affect human populations (Fair et al. 2019). Data integration platforms combining environmental, wildlife, and human monitoring offer opportunities for comprehensive assessment across domains (Houde et al. 2011).

### Policy innovations and regulatory approaches

The complexity and scope of PFAS contamination have driven innovations in regulatory approaches increasingly reflecting One Health principles (Cousins et al. 2020; Kwiatkowski et al. 2020).

#### Class-based regulation and essential use frameworks

The development of class-based regulatory approaches represents a significant innovation addressing challenges posed by the vast number of PFAS compounds (Kwiatkowski et al. 2020). Due to their large number and structural similarities, traditional approaches assessing compounds individually are inadequate for PFAS (Idowu et al. 2025). Class-based approaches recognize that PFAS share common properties including persistence, mobility, and bioaccumulation potential justifying treatment as a class (Kwiatkowski et al. 2020). Essential use frameworks require clear criteria for determining which PFAS applications are truly necessary and cannot be replaced with safer alternatives (Cousins et al. 2019). These criteria must consider technical performance requirements and alternative availability. Variations in PFAS toxicity and environmental fate have led to a division between substance-specific risk assessments and comprehensive class-based regulations. Those favoring a group approach prioritize the precautionary principle to avoid replacing banned substances with similar hazardous alternatives, whereas others contend that aggregate regulation ignores distinct chemical risks. Recent policy frameworks often combine these strategies by strictly controlling well-documented compounds like PFOA and PFOS while implementing broader restrictions for the entire class, except where uses are deemed essential (Cousins et al. 2020; ECHA, 2023).

#### International cooperation and governance

The global nature of PFAS contamination requires international cooperation and coordination to address sources, manage contamination, and protect health across borders (Stockholm Convention, 2023). The Stockholm Convention provides a framework for international cooperation, although its compound-by-compound approach limits effectiveness. Regional cooperation initiatives provide frameworks for coordinated monitoring and assessment in vulnerable regions.

The integration of environmental justice principles into PFAS policy represents an important evolution recognizing disproportionate impacts on vulnerable communities (Brunn et al. 2023). Environmental justice integration requires considering the distribution of environmental benefits and burdens across communities and ensuring meaningful participation in decision-making.

### Knowledge gaps and research needs

Critical knowledge gaps and research priorities essential for advancing One Health PFAS management are presented in Table 3. These priorities emphasize transdisciplinary collaboration to resolve uncertainties in mixture toxicology, long-term effects, climate interactions, and remediation technologies.

**Table 3:** Critical knowledge gaps and research priorities for one health PFAS management

Research domain	Specific knowledge gaps	Research priorities	Expected one health benefits
Mixture effects and risk assessment	Limited understanding of mixture toxicity, non-monotonic dose responses, cumulative effects of multiple PFAS compounds	Develop mixture assessment methods, validate computational approaches (QSAR, read-across), conduct long-term mixture studies	Improved risk assessment for realistic exposure scenarios, better protection across species
Long-term and multigenerational effects	Insufficient data on chronic effects, transgenerational impacts, epigenetic modifications	Establish long-term cohort studies, investigate epigenetic mechanisms, conduct multigenerational wildlife studies	Understanding of lifetime and intergenerational health impacts, improved protection of vulnerable populations
Climate change interactions	Unknown effects of changing temperature and precipitation on PFAS fate and transport	Study climate impacts on PFAS behavior, investigate Arctic vulnerability, assess extreme weather effects	Adaptation strategies for changing environmental conditions, protection of climate-vulnerable ecosystems
Analytical chemistry	Limited capability to detect unknown PFAS, transformation products, total organofluorine burden	Develop non-targeted analytical methods, identify transformation pathways, improve detection limits	Comprehensive exposure assessment, early detection of emerging compounds, better environmental monitoring
Ecosystem-level effects	Insufficient understanding of population and community impacts, food web effects, ecosystem services	Conduct long-term ecosystem studies, develop food web models, assess microbial community effects	Protection of ecosystem function and services, understanding of ecological carrying capacity
Remediation technology	Limited effectiveness of current technologies, lack of in-situ treatment options, unknown ecological impacts	Develop destruction technologies, assess ecological impacts of remediation, improve cost-effectiveness	Effective contamination cleanup with minimal ecological disruption, restoration of environmental services
Environmental justice	Limited understanding of cumulative impacts, differential vulnerability, community-specific exposure patterns	Conduct cumulative risk assessments, investigate social determinants of exposure, develop community-based interventions	Equitable protection across populations, reduced health disparities, community empowerment
Alternatives assessment	Incomplete evaluation of PFAS alternatives, potential for regrettable substitutions, lifecycle impacts	Develop alternatives assessment frameworks, investigate substitute safety, conduct lifecycle analyses	Prevention of regrettable substitutions, sustainable chemistry development, innovation in safer technologies

### Comprehensive mixture effects and risk assessment

The assessment of mixture effects represents one of the most significant challenges, as environmental samples typically contain complex mixtures of dozens or hundreds of PFAS compounds (Bil et al. 2021; Goodrum et al. 2021). Current approaches based on individual compounds are inadequate for mixture exposure reality. Non-monotonic dose–response relationships and low-dose effects represent additional challenges, as traditional linear models may not adequately characterize health risks (Fenton et al. 2021).

### Long-term and multigenerational effects

The long environmental persistence and biological half-lives of PFAS create opportunities for long-term and multigenerational effects, which are difficult to study using traditional approaches (Fenton et al. 2021). Epidemiological studies are limited by relatively recent recognition of widespread exposure and long latency periods for chronic diseases (Boston et al. 2025; Wee and Aris 2023). As evidence suggests PFAS exposure can cause epigenetic modifications transmitted to subsequent generations, transgenerational effects represent a particularly important research need (Abdulkadir et al. 2025; Everson et al. 2025; Kebieche et al. 2025).

### Omics approaches and emerging biomarkers

Advances in transcriptomics, metabolomics, proteomics, and epigenomics are uncovering novel mechanisms of PFAS toxicity across species. These omics-based studies enable the identification of sensitive biomarkers capable of detecting early molecular perturbations before clinical effects become apparent. Incorporating such approaches into PFAS research could significantly improve exposure assessment, cross-species comparisons, and the development of predictive models for health outcomes. (Beale et al. 2022; Zhou et al. 2025).

However, the translation of omics findings into validated biomarkers remains a major challenge. Results are often heterogeneous across studies and lack standardization in methodologies and reporting. Moreover, the integration of multi-omics datasets requires robust computational frameworks and systems biology approaches to disentangle causal pathways from adaptive responses. Addressing these gaps will be crucial to establishing reliable biomarker panels that can support human biomonitoring, ecological risk assessment, and ultimately inform regulatory decision-making.

### Climate change interactions

Climate change may alter PFAS environmental fate and transport in ways not yet fully understood but with significant implications for exposure and risk assessment (Liu and Mejia Avendaño, 2013; Weber et al. 2017; Zhou et al. 2024). Rising temperatures may increase volatilization and atmospheric transport of certain PFAS compounds (Weiwei et al. 2024). Arctic environments face specific vulnerabilities from climate-PFAS interactions, as changing ice conditions may alter partitioning and bioavailability in marine systems (Lohmann et al. 2024).

### Analytical chemistry and unknown compounds

Despite significant progress, major analytical challenges remain in detecting and quantifying the vast diversity of PFAS compounds in environmental and biological samples (Cousins et al. 2020). Current targeted methods typically cover only a few dozen analytes, representing a small fraction of the thousands of PFAS structures known or suspected to be present (Thompson et al. 2024). This limitation hampers accurate exposure assessment and toxicological evaluation, as many overlooked compounds may contribute substantially to overall risk. The development of non-targeted and suspect screening approaches is, therefore, a rapidly expanding research frontier, leveraging high-resolution mass spectrometry (HRMS), advanced chromatographic separations, and machine learning–based data processing (Gonzalez de Vega et al. 2021). Such methodologies enable the detection of unknown PFAS congeners, transformation products, and short-chain analogues that are often missed by conventional assays. However, critical challenges persist, including the lack of reference standards, the complexity of isomeric mixtures, and the need for harmonized workflows to ensure reproducibility and comparability across laboratories. Addressing these analytical gaps is essential not only for improving environmental monitoring but also for strengthening human biomonitoring, food safety assessments, and regulatory frameworks that depend on accurate and comprehensive PFAS identification.

In addition to these methodological constraints, the high cost associated with advanced analytical techniques, particularly chromatography coupled with mass spectrometry and high-resolution mass spectrometry, poses a significant barrier. These platforms require not only substantial financial investment for acquisition and maintenance but also highly trained personnel for reliable operation and data interpretation. In many emerging countries, the limited availability of such infrastructure further restricts the implementation of cutting-edge PFAS analysis, resulting in considerable knowledge gaps in the literature and an underrepresentation of data from regions that are equally, if not more, vulnerable to environmental contamination. Bridging these disparities is critical to achieving a more globally balanced understanding of PFAS exposure and risks.

### Ecosystem-level effects and food web dynamics

Understanding ecosystem-level effects requires research examining impacts on populations, communities, and ecosystem function beyond individual organism effects (Fair et al. 2019; Houde et al. 2011). Food web modeling approaches incorporating PFAS bioaccumulation and biomagnification can provide tools for predicting ecosystem-level effects and assessing management strategy effectiveness (Kelly et al. 2024).

### Remediation technology development

The development of effective, scalable, and economically feasible remediation technologies remains one of the most pressing challenges in addressing PFAS contamination, requiring strong interdisciplinary collaboration that integrates chemistry, engineering, toxicology, and environmental sciences (Ross et al. 2018; Weber et al. 2017). Current treatment strategies, such as activated carbon adsorption, ion exchange resins, and high-pressure membrane filtration, are effective for certain PFAS but are often limited by high operational costs, incomplete removal, and difficulties in managing concentrated waste streams. Moreover, these technologies generally transfer contaminants from one medium to another rather than achieving complete destruction.

In-situ remediation approaches that can treat contamination directly in soils, sediments, and groundwater without excavation are particularly needed for large and diffuse contaminated sites (Mahinroosta and Senevirathna 2020). Promising avenues include electrochemical oxidation, advanced photocatalysis, plasma-based destruction, and thermal treatments, though many of these methods remain at the bench or pilot scale. Bioremediation, while still underexplored, has shown preliminary evidence of microbial pathways capable of degrading certain PFAS analogues, representing an innovative but challenging frontier (Shahsavari et al 2021).

Critical barriers include the chemical stability of PFAS due to strong C–F bonds, the diversity of PFAS structures that resist a one-size-fits-all treatment, and uncertainties regarding the formation of potentially toxic transformation products during partial degradation. Developing life-cycle assessments and techno-economic analyses is equally important to evaluate the sustainability, scalability, and environmental trade-offs of emerging technologies. Addressing these challenges will require harmonized international research efforts and public–private partnerships to accelerate the transition from laboratory innovations to field-scale applications.

## Discussion and conclusions

This comprehensive review demonstrates that PFAS contamination represents the quintessential One Health challenge of the modern era, demanding unprecedented levels of integration and collaboration across traditional disciplinary boundaries (Adamopoulos et al. 2025; Destoumieux-Garzón et al. 2018; Lerner and Berg 2017; Wang et al. 2024). The evidence synthesized through this One Health lens reveals several critical conclusions that fundamentally reshape our understanding and response to PFAS contamination.

### Key findings and implications

PFAS contamination exemplifies how industrial decisions made decades ago continue to affect environmental and health outcomes across all domains of the One Health paradigm (Shurson 2025). The extreme persistence of these compounds means that contamination patterns established during peak production years will continue affecting human populations, wildlife communities, and ecosystem function for generations. This legacy contamination creates shared vulnerabilities that cannot be addressed through traditional single-domain approaches. The remarkable consistency of PFAS-induced health effects across diverse species provides compelling evidence for shared toxicological mechanisms that transcend species boundaries (Fenton et al. 2021). Immunotoxicity, reproductive impairment, developmental toxicity, and liver dysfunction occur across species with similar dose–response relationships, validating the use of wildlife and laboratory animal data to inform human health protection while demonstrating that effective intervention strategies must protect all affected species simultaneously (Bline et al. 2024; DeWitt et al. 2006, 2009).

PFAS contamination reveals stark patterns of environmental injustice, with vulnerable human populations and sensitive ecosystems bearing disproportionate burdens from industrial activities (Fenton et al. 2021). The intersection of PFAS contamination with other environmental and social stressors creates cumulative impacts that exceed the sum of individual exposures, requiring intervention strategies that address both underlying inequities and direct contamination sources.

### Regulatory and scientific frontiers

The challenges posed by thousands of structurally related PFAS compounds have driven important innovations in regulatory approaches, including class-based regulation, essential use frameworks, and integrated risk assessment methods (Lendewig et al. 2025). However, the global nature of PFAS contamination and continuing development of new compounds require unprecedented levels of international coordination.

Current remediation technologies, analytical methods, and risk assessment approaches are inadequate for addressing the scope and complexity of PFAS contamination (Bayode et al. 2024). Significant advances are needed in destruction technologies, non-targeted analytical methods, mixture risk assessment, and long-term health effects research, requiring sustained investment in transdisciplinary research.

Importantly, these needs are even more pronounced in low- and middle-income countries, where limitations in analytical infrastructure, biomonitoring capacity, and regulatory oversight severely constrain the ability to detect contamination, characterize toxic effects, and implement effective monitoring strategies. This lack of region-specific evidence hampers comprehensive exposure assessments and prevents the establishment of reliable baselines for evaluating ecological and human health risks. The absence of locally generated data in emerging economies contributes directly to the lack of regulatory frameworks, perpetuating an underrepresentation of these regions in the global discourse on PFAS risk management and further delaying the implementation of effective mitigation strategies. Strengthening global cooperation, technology transfer, and capacity building will therefore be essential to ensure that all regions, particularly those most vulnerable, are equipped to confront the PFAS crisis (Souza et al. 2022).

### Transformation of environmental health practice

The PFAS challenge illustrates limitations of traditional environmental health approaches that treat human health, animal health, and environmental protection as separate concerns. Effective PFAS management requires a transformation of environmental health practice to embrace One Health principles, recognizing the fundamental interconnectedness of all health domains and developing integrated solutions that generate co-benefits across domains (Ogunseitan 2022; Oliveira and Gebreyes 2022).

### Prevention and urgency

The extreme persistence and global distribution of PFAS demonstrate that preventing contamination is far more effective and economically efficient than attempting remediation after contamination has occurred. This lesson has profound implications for the regulation of other persistent chemicals and the development of sustainable chemistry approaches, considering environmental and health impacts from the earliest stages of chemical development.

The continuing production and use of PFAS compounds, combined with the irreversible nature of environmental contamination, create an urgent need for coordinated action transcending traditional institutional boundaries (Evich et al. 2022). Every day of delay in implementing comprehensive PFAS management strategies results in additional contamination that will persist essentially forever.

### Path forward

The path forward requires unprecedented collaboration among scientists, policymakers, industry, and communities to develop and implement solutions addressing the root causes of contamination, while protecting the most vulnerable populations and ecosystems. Such collaboration must be global in scope and maintained over decades, reflecting both the persistence of PFAS in the environment and the intergenerational nature of their health impacts.

The ultimate success of PFAS management efforts will be measured not only by reductions in environmental concentrations and health effects, but also by the ability to prevent similar contamination problems in the future through the development of sustainable chemistry approaches and regulatory systems that adequately protect environmental and human health (Suffill et al. 2024).

The One Health framework provides the conceptual foundation for this transformation, emphasizing that the health of humans, animals, and the environment is inextricably linked. Sustainable solutions must protect all three domains simultaneously. The application of One Health principles to PFAS management demonstrates both the potential for more effective environmental health protection and the institutional changes needed to realize this potential (Shurson 2025).

## Data Availability

Not applicable.
